# *Ruta angustifolia* Pers. (Narrow-Leaved Fringed Rue): Pharmacological Properties and Phytochemical Profile

**DOI:** 10.3390/plants12040827

**Published:** 2023-02-13

**Authors:** Christian Bailly

**Affiliations:** 1 OncoWitan, Scientific Consulting Office, Wasquehal, F-59290 Lille, France; 2 Institut de Chimie Pharmaceutique Albert Lespagnol (ICPAL), Faculté de Pharmacie, University of Lille, 3 rue du Professeur Laguesse, BP-83, F-59006 Lille, France; 3 CNRS, Inserm, CHU Lille, UMR9020-U1277—CANTHER—Cancer Heterogeneity Plasticity and Resistance to Therapies, University of Lille, F-59000 Lille, France

**Keywords:** alkaloids, anticancer agents, furocoumarins, *Ruta angustifolia*, *Ruta* species

## Abstract

The genus *Ruta* in the family *Rutaceae* includes about 40 species, such as the well-known plants *R. graveolens* L. (common rue) or *R. chalepensis* L. (fringed rue), but also much lesser-known species such as *R. angustifolia* Pers. (narrow-leaved fringed rue). This rue specie, originating from the Mediterranean region, is well-distributed in Southeast Asia, notably in the Indo-Chinese peninsula and other territories. In some countries, such as Malaysia, the plant is used to treat liver diseases and cancer. Extracts of *R. angustifolia* display antifungal, antiviral and antiparasitic effects. Diverse bioactive natural products have been isolated from the aerial parts of the plant, notably quinoline alkaloids and furocoumarins, which present noticeable anti-inflammatory, antioxidant and/or antiproliferative properties. The present review discusses the main pharmacological properties of the plant and its phytoconstituents, with a focus on the anticancer activities evidenced with diverse alkaloids and terpenoids isolated from the aerial parts of the plant. Quinoline alkaloids such as graveoline, kokusaginine, and arborinine have been characterized and their mode of action defined. Arborinine stands as a remarkable inhibitor of histone demethylase LSD1, endowed with promising anticancer activities. Other anticancer compounds, such as the furocoumarins chalepin and rutamarin, have revealed antitumor effects. Their mechanism of action is discussed together with that of other bioactive natural products, including angustifolin and moskachans. Altogether, *R. angustifolia* Pers. presents a rich phytochemical profile, fully consistent with the traditional use of the plant to treat cancer. This rue species, somewhat neglected, warrant further investigations as a medicinal plant and a source of inspiration for drug discovery and design.

## 1. Introduction

The Rutaceae family covers a very large group of flowering plants with a worldwide distribution (order: Sapindales). They are commonly known as the citrus family, with about 160 genera, including economically important ones such as *Citrus sinensis* (L) Osbeck (orange), *C. limon* (L) Osbeck (lemon), *C. paradisi* Mcfad. (grapefruit), and *C. aurantifolia* Linn. (lime) [1]. The family also includes less-known species, such as the cultivar *C. aurantium* (called ‘Changshan-huyou’ in China), a hybridization of *C. grandis* Osbeck and *C. sinensis* Osbeck, which has been cultivated for hundreds of years in China [2]. The peel or fruits of different varieties of *Citrus* provide raw materials for some traditional Chinese medicine (TCM), such as *C. maxima* (Burm.) Merr., *C. reticulata* Blanco, *C. wilsonii* Tanaka, and several others [3,4,5]. The Rutaceae family also includes less-known species, notably the *Ruta* genus, which comprises about 40 different species largely distributed in the Mediterranean region. The genus includes well-studied species such as *R. chalepensis* L., *R. graveolens* L., and *R. montana* L., used to manufacture essential oils and to prepare traditional medicines for the treatment of lung diseases and microbial infections [6]. A lesser-known medicinal plant of the family is *Ruta angustifolia* Pers., originally found in Southern Europe and North Africa, but now largely distributed in many countries, notably in Asia. The plant is known as ‘godong minggu’ (Javanese) or ‘daun inggu’ (Sundanese) in Indonesia [7], ‘garuda’ or ‘sadal’ in Malaysia, ‘luru’ in Vietnam (cửu lý hương), and ‘rue’ (specifically the narrow-leaved fringed ‘rue’) in English and French [8].

**Figure 1:**
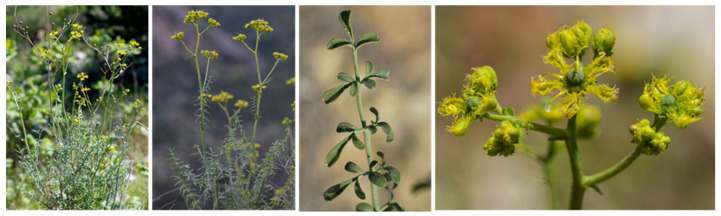
The plant *Ruta angustifolia* Pers. (FloresAlpes, https://www.florealpes.com) accessed on 17 December 2022.

*Ruta angustifolia* Pers. generally grows up in the mountains to an elevation of 1000 m above sea level, notably in the subtropical biome where soils are relatively warm. This tall subshrub (0.4–1 m height), with a self-supporting growth form, presents a slender woody stem and light green leaves (Figure 1). The plant flowers normally between April to July, and the yellow flowers give off a very strong fetid odor. The plant reproduces via seeds and also grows from stem cuttings. Genetically, *R. angustifolia* Pers. has similarities with *R. graveolens* L. and *R. chalepensis* L. [8]. However, various natural products of pharmacological interest have been isolated from this *Ruta* species, and specific medicinal effects have been evidenced as well. An overview of the medicinal properties of *R. angustifolia* Pers. and isolated natural products is presented here. This specific species has been less investigated than other *Ruta* [9]. It is nevertheless a useful medicinal plant and an under-exploited source of bioactive products.

## 2. Pharmacological Activities of *R. angustifolia* Extracts

*Ruta* species have long been used for the treatment of various human diseases. The so-called *Corpus Hippocraticum* (a collection of 62 treatises written between the 5th century BCE and the 2nd century BCE), which is arguably the most ancient systematic record of medical practice of the Mediterranean world, refers to medical applications of these plants. Hippocratic physicians considered *Ruta* plants as a remedy suitable for diseases in women and as an effective *pharmakon* (the Greek word for drug) in the cure of pulmonary affections [10]. Today, the common rue (*Ruta graveolens* L.) remains an important plant in traditional medicine in many countries [11], notably utilized in the treatment of female menstrual diseases [12]. There are less reports about the use of *R. angustifolia* Pers. but nevertheless, in Indonesia the plant is apparently used as a decoction to cure cramps, flatulence, fever as well as for liver disease and jaundice [13]. Another publication mentions the traditional use of *R. angustifolia* for treating ear infections, boils and bruises [14].

Extracts of *R. angustifolia* have revealed antifungal but no antibacterial activities. A total methanolic extract from a specimen collected in Malaysia was shown to exert a modest antioxidant effect but no antibacterial effect in vitro [15]. Similarly, an essential oil made from a plant collected in Algeria showed no antibacterial activities, whereas minor activities were observed with other *Ruta* species [16]. The lack of antibacterial effect is somewhat surprising because the plant has been clearly shown to synergize with antibiotics such as erythromycin or vancomycin [14]. *R. angustifolia* contains various quinoline alkaloids with antimicrobial activities (see below). These quinoline alkaloids are perhaps not sufficiently abundant in the prepared extracts to reveal an antibacterial activity with those extracts.

**Figure 2:**
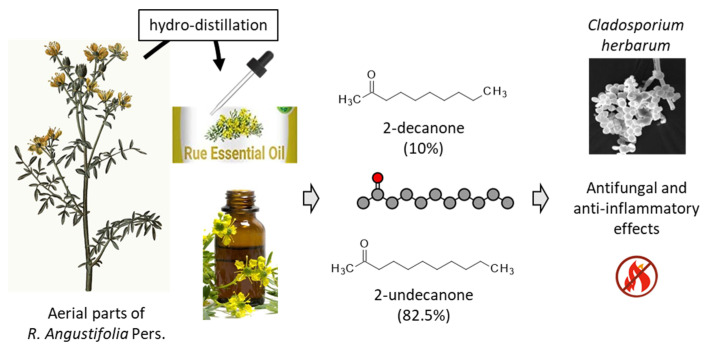
An essential oil manufactured from the aerial parts of *Ruta angustifolia* Pers. contained essentially 2-ketone derivatives, principally 2-undecanone and 2-decanone, which display antifungal and anti-inflammatory properties [6,16].

Marked antifungal effects have been reported with essential oils prepared from different *Ruta* species, including *R. angustifolia*. The essential oil from this later species revealed a marked activity against the two filamentous fungi *Aspergillus fumigatus* and *Cladosporium herbarum*, with minimum inhibitory concentration (MIC) < 3.5 and 4.7 µg/mL, respectively [16]. 2-Ketone derivatives were abundant in the oil (95.6%), notably 2-undecanone, which is a classical antifungal and anti-inflammatory agent (Figure 2). This natural ketone is commonly found in essential oils from various *Ruta* species [6,17,18]. In addition, alkaloids contribute to the antifungal activity of the plant, notably arborinine and graveoline present in the leaves of *R. angustifolia*, as detailed below [19]. Recently, an activity has been mentioned also using an ethanolic leave extract of *R. angustifolia* on a preformed biofilm of *C. albicans*, but no quantitative information was reported [20].

Antiviral effects have been reported with leave extracts against an experimental strain (J6/JFH1-P47) of hepatitis C virus (HCV). This virus is responsible for chronic hepatitis, liver cirrhosis and hepatocellular carcinoma in humans. A potent activity was observed with a dichloromethane extract (IC_50_ = 1.6 µg/mL), from which several active compounds were isolated, including the alkaloid pseudane IX (IC_50_ = 1.4 µg/mL) and the dihydrofuranocoumarin derivative chalepin (IC_50_ = 1.6 µg/mL), both described below [21]. Diverse secondary metabolites have been isolated from the plant leaves, depending on the polarity of the solvent and processes used. The extraction yield is usually better with methanol (polar) compared to ethyl acetate or hexane (apolar), but bioactive compounds can be found in different fractions. A chloroform extract has been shown to exert a significant antiproliferative activity against cancer cells in vitro (IC_50_ = 8.8 µg/mL with A549 lung cancer cells), mostly due to the presence of chalepin, chalepensin, and a few other products (see below) [13].

Extracts of *Ruta* species have been tested as antiparasitic and insecticidal/larvicidal agents as well. Many interesting bioactive compounds have been isolated and characterized, notably a variety of anti-inflammatory, anti-proliferative or cell-protective compounds [9]. However, to our knowledge, no major activity against human parasites has been reported specifically with *R. angustifolia* extracts. There is a minor mention of the traditional use of the plant branches to control insect propagation by farmers in the area of Arribes del Duero (Salamanca, Spain) [22]. Moreover, a recent study described the biocidal effect of a propyl ester of ergosterol, isolated from an acetonic extract of *R. angustifolia* leaves. The ester has revealed a larvicidal action against the cotton leaf worm *Spodopetra littoralis* (Boisd.) [23]. The insecticidal and antiparasitic activities of the plant should be further explored. The plant contains volatile substances, such as 2-decanone, 2-nonanone and 2-undecanone, found also in other rue species and which contribute to the repellent activity of the shrub [24,25]. These three products, found for example in *R. chalepensis*, have been characterized as anthelmintic compounds [26]. They are also present in *R. angustifolia* [6]. Extracts or essential oils from *R. angustifolia* could be used to reduce damages caused by phytoparasitic nematodes, as it is the case with other *Ruta* species [27,28].

## 3. Bioactive Phytochemicals from *R. angustifolia* Pers.

### 3.1. Quinoline Alkaloids

**Figure 3:**
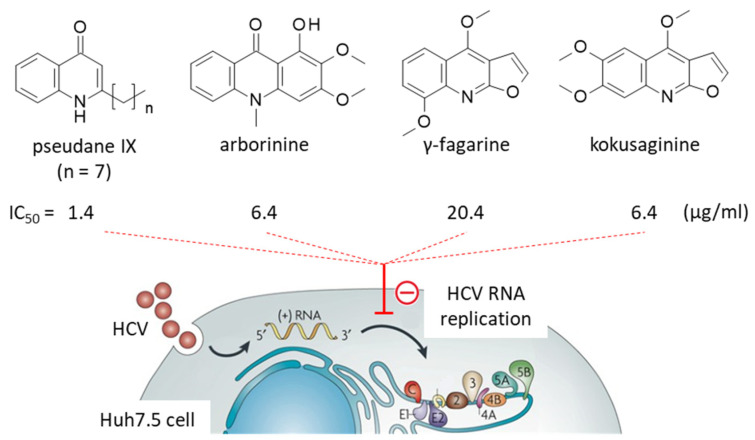
Inhibition of HCV RNA replication by the alkaloids pseudane IX, γ-fagarine, arborinine, and kokusaginine isolated from *Ruta angustifolia* Pers. The most potent alkaloid is pseudane IX which inhibited replication more potently than the reference product ribavirin (IC_50_ = 1.4 and 2.8 µg/mL, respectively) [21].

The presence of specific alkaloids in *Ruta* species was underlined more than 60 years ago with the isolation of the first furanocoumarin and furoquinoline alkaloids, such as dictamnine and evolitrine [29]. But these products were isolated mainly from *R. graveolens* L. and *R. montana* Mill. [30]. The presence and bioactivities of alkaloids in *R. angustifolia* Pers. was unambiguously established much later, in 2014 with the isolation of the quinolinone alkaloid pseudane IX and the characterization of its activity against the HCV virus [21]. Pseudane IX was found to inhibit HCV replication, predominantly at the post-entry step (IC_50_ = 3.0 µg/mL post-entry versus 11.5 µg/mL at the entry step, in Huh7.5 cells infected with HCV). The compound functions as an inhibitor of HCV RNA replication and viral protein synthesis. In the viral system used (HCV strain J6/JFH1-P47), pseudane IX turned out to be more potent that the reference product ribavirin (IC_50_ = 1.4 and 2.8 µg/mL, respectively) and the alkaloid only exerted a modest cytotoxic action (CC_50_ = 26 µg/mL). In this study, three other quinoline alkaloids were isolated, namely γ-fagarine, arborinine, and kokusaginine (IC_50_ = 20.4, 6.4 and 6.4 µg/mL, respectively), but they were found to be less active against HCV than pseudane IX [21] (Figure 3). This work shed light on pseudane alkaloids, a small series of under-exploited compounds, found in diverse plants and/or microorganisms. Pseudane IX (with a C9 alkyl side chain at position 2 on the quinolinone) displays antiviral effects, and pseudane VII (with a C7 alkyl side chain at the same position), isolated from a *Pseudoalteromonas* species, display marked anti-inflammatory activities [31,32,33]. Pseudanes are 2-n-alkyl-quinolin-4-one initially isolated from the Gram-negative aerobic bacillus *Pseudomonas aeruginosa* [34]. They have been little studied thus far, but they would deserve additional investigations to better comprehend their activities and molecular targets. They can be obtained from natural sources, or synthesized. Recent procedures have been described to synthesize pseudanes and related 4-(1*H*)-quinolones such as graveoline and graveolinine [35,36].

The case of the furoquinoline alkaloids, kokusaginine merits a mention because the compound, found in diverse plants, has been shown to inhibit tubulin formation and assembly. It binds to the colchicine site on tubulin, thereby inhibiting the growth of breast cancer cells, including multidrug resistant sublines such as MCF-7/ADR and MDA-MB-231/ADR [37]. Kokusaginine also displays modest cholinesterase inhibitory activities (IC_50_ = 41.46 and 70.24 µg/mL against butyrylcholinesterase and acetylcholinesterase, respectively) [38,39]. The compound has been used as a starting point to the design of antiparasitic molecules active against *Trypanosoma cruzi* responsible for the Chagas disease [40]. Kokusaginine is an isomer of skimmianine and a close analogue of dictamnine, both furoquinolines commonly found in *Ruta* species and endowed with anticancer properties [41,42,43]. There is a sound basis to investigate further the anticancer properties of kokusaginine and related furoquinoline alkaloids.

**Figure 4:**
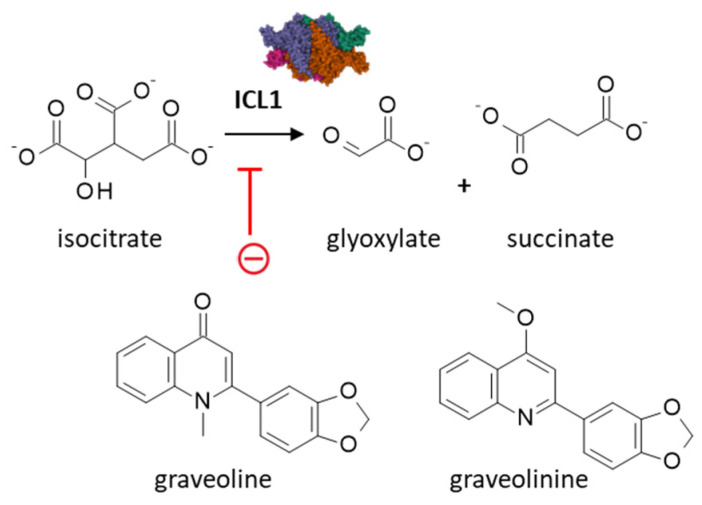
Graveoline is an inhibitor of isocitrate lyase 1 (ICL1) from the fungus *Candida albicans* [19]. The enzyme catalyzes the cleavage of isocitrate to succinate and glyoxylate. The analogue graveoline is not active against ICL1 but displays antiangiogenic properties. These two quinoline alkaloids can be found in *Ruta* species, including *R. angustifolia* Pers.

Many quinoline alkaloids have been isolated from *Ruta* species, such as dictamnine, pteleine, skimmianine, rutacridone, maculosidine, graveoline, graveolinine [44]. The quinolone alkaloid graveoline, initially isolated from the common rue (*Ruta graveolens*) [11], is a major phytotoxin from *Ruta* plants [45]. It is found in many *Ruta*, including the leaves of *R. angustifolia* [14,19]. In this specie, graveoline (Figure 4) has been characterized recently as an antifungal agent inhibiting the expression of the enzyme isocitrate lyase 1 (ICL1) in the fungus *Candida albicans*, as observed with the reference antifungal product fluconazole [19]. However, the mechanism of action of this compound is multifactorial because it has been characterized as an inhibitor of photosynthesis [46] and as an antitumor agent capable of inducing production of reactive oxygen species (ROS) in cancer cells and triggering both apoptotic and autophagic cell death [47]. The compound is easily metabolized in the liver, with up to 12 metabolites identified [48]. The derivative graveolinine (Figure 4) displays weaker anti-angiogenic activity than the parent compound graveoline [49] but it can serve as a starting point for the design of anti-Alzheimer agents [50]. The compound has been shown to bind to the serotonin 5-HT2B receptor and to inhibit weakly the cyclooxygenase 2 enzyme (COX2, 79% inhibition at 150 µM) [51].

**Figure 5:**
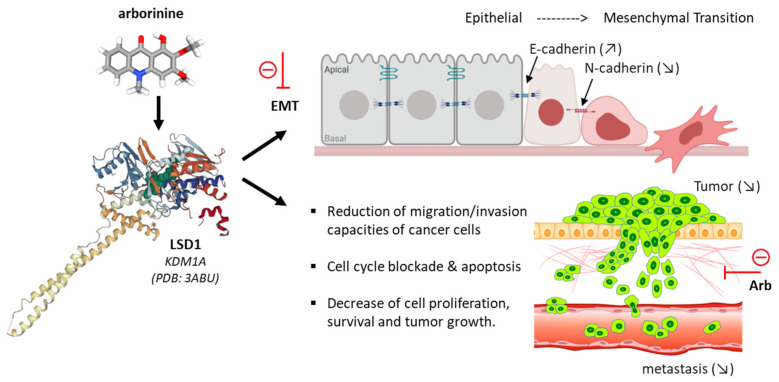
The structure of arborinine and its action as an inhibitor of histone lysine-specific demethylase 1 (LSD1), which is frequently overexpressed in cancer cells. Via this process, arborinine impairs the EMT dynamic (epithelial–mesenchymal transition) and reduces aggressivity of cancer cells in terms of survival, growth, dissemination and metastasis. Administered orally, arborinine can reduce tumor growth in a xenograft model of gastric cancer in mice [60].

A quinolin-4-one moiety is also present in arborinine, another alkaloid found in *R. angustifolia* (Figure 3). As mentioned above, this tricyclic compound can inhibit HCV replication, but it is less potent than pseudane IX [21], and it contributes to the antifungal activity of the plant extract [19]. Arborinine can be isolated from diverse plants, including Rutaceae such as *Vepris trichocarpa* and *Vepris teva* [52,53], *Araliopsis soyauxii* [54] and different *Ruta* species [55]. Interestingly, the acridone alkaloid has been shown to exert marked antitumor activities. It inhibits dose-dependently the proliferation of several cancer cell lines [56,57], triggers cell cycle arrests, blocks cancer cell migration, and induces apoptosis [58,59]. It displays a sub-micromolar activity against drug-resistant SGC-7901 gastric cancer cells resistant to adriamycin (SGC-7901/ADR: IC_50_ = 0.24 μM) or to vincristine (SGC-7901/VCR: IC_50_ = 1.09 μM) [60]. Arborinine has been characterized as a selective and reversible inhibitor of histone lysine-specific demethylase 1 (LSD1), an enzyme frequently overexpressed in cancer cells. LSD1 is a key enzyme, implicated in the epithelial-mesenchymal transition (EMT) and in tumor progression (Figure 5). Arborinine has the capacity to repress EMT in cancer cells, modulating the expression of specific markers (upregulation of E-cadherin, downregulation of N-cadherin and vimentin). The blockade of LSD1 with arborinine induces a dose-dependent accumulation of methylated histone H3 in cells (H3K4me1, H3K9me1, H3K9me2), thereby inducing inhibition of cancer cell migration, invasion and proliferation.

**Figure 6:**
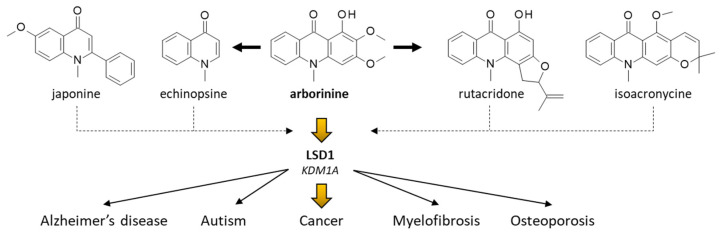
Structural analogy between arborinine and other bi, tri or tetracyclic alkaloids with a 1-methylquinolin-4-one (echinopsine) unit. These compounds represent potential LSD1 inhibitors, which may be used to treat diverse human diseases, such as those indicated.

Remarkably, the compound was shown to exert a significant antitumor effect in vivo, in two xenografted murine model of gastric cancer (with SGC-7901 cells sensitive or resistant to adriamycin). At the oral dose of 40–80 mg/kg, arborinine reduced tumor growth in mice, without causing any apparent toxicity [60]. By the same token, very recently arborinine has been shown to suppress ovarian cancer development through inhibition of LSD1. The blockade of LSD1 leads to an increased expression of methylated histone H3K4m1 in SKOV3 ovarian cancer cells, thereby reducing the migration and invasion capacities of the cells due to a blocked EMT [61]. The effect is not specific to ovarian cancer cells because the LSD1 enzyme (also known as lysine (K)-specific demethylase 1A, or KDM1A) is present and frequently overexpressed in many cancer cell types. Recently, it was demonstrated that arborinine can block LSD1/KDM1A activity in clear-cell renal cell carcinoma ccRCC cell lines, sensitive or resistant to the kinase inhibitor sorafenib. In this case again, the compound blocked cell migration and invasion, cell cycle progression, and induced apoptosis [62]. A molecular modeling analysis suggested that arborinine could bind directly to the active site of LSD1, and blocks downstream signaling activities, notably the expression of the ubiquitin-conjugating enzyme E2O (UBE2O), an important protein for cancer cell survival and proliferation [62]. The observation that arborinine represses LSD1/UBE2O signaling in ccRCC opens novel perspectives for research. Firstly, in terms of drug design, it opens a field of research to discover other lysine demethylase inhibitors with an acridone or quinolin-4-one moiety. There exist many naturally-occurring analogues of arborinine, such as evoxanthine, rutacridone, quinolactacins A-C, (iso)acronycine, melicopicine and others tri/tetracyclic products. Bicyclic products derived from echinopsine shall be considered as well, notably the panel of 1-methylquinolin-4-one alkaloids such as eduline, japonine and others (Figure 6). Secondly, in terms of clinical applications, LSD1/KDM1A is aberrantly overexpressed in several cancer types, with different LSD1 inhibitors currently developed to treat solid tumors and hematological malignancies [63,64,65]. The antitumor activity of arborinine shall be investigated using a variety of experimental models, to define the optimal conditions and the most suitable applications in humans. Thirdly, outside oncology, LSD1/KDM1A is an enzyme of interest to treat pathologies such as myelofibrosis [66], autism [67] and other diseases [68]. The targeting of LSD1 with arborinine may have broad therapeutic implications; recently, Merck paid US$1.35 billion for LSD1 inhibitors [69]. Parenthetically, another mechanism of action has been evoked with arborinine: the binding to heme leading to a reduction of oxidative stress and inflammation [70]. However, the heme binding affinity is weak (K_a_ = 3.9 × 10^4^ M^−1^) compared to the level of LSD1 inhibition. Nevertheless, arborinine exhibits anti-inflammatory activities. It is able to reduce the production of nitric oxide (NO) in activated macrophages [71].

### 3.2. Furocoumarins

**Figure 7:**
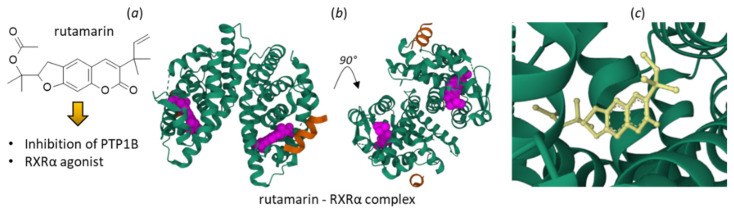
(**a**) Structure of rutamarin and (**b**) its binding to human retinoic X receptor alpha (RXRα). The alkaloid (purple) is bound to the protein dimer (green), in the presence of a SRC1 peptide (orange). (**c**) A close-up view of rutamarin in the RXRα active site (from PDB structure 3PCU) [78].

Rutamarin is an anticancer furocoumarin derivative isolated from *R. angustifolia* Pers. [72] and other *Ruta* species [73]. The compound is moderately cytotoxic to colorectal adenocarcinoma HT29 cells (IC_50_ = 5.6 μM), and induces cell cycle perturbations and caspase-dependent apoptosis [73]. At the molecular level, rutamarin is believed to function as a catalytic inhibitor of human topoisomerase II (not a classical Topo II poison), binding to the ATPase domain of the enzyme, thereby blocking DNA replication [74]. This mechanism accounts for both the anticancer and antiviral activities of rutamarin, and probably for its antiprotozoal activity as well [75]. Rutamarin inhibits DNA replication of Epstein-Barr virus (EBV) (IC_50_ = 7.0 μM) [76,77] and Kaposi’s sarcoma-associated herpesvirus (KSHV) (IC_50_ = 1.12 μM), at least the (+)-enantiomer [74]. Beyond TopoII, two additional targets for rutamarin have been proposed on the basis of a molecular modeling analysis: the protein tyrosine phosphatase 1B (PTP1B) and the retinoid X receptor α (RXRα). Rutamarin would be an inhibitor of PTP1B and a RXRα agonist. The molecular models have been validated experimentally. Rutamarin effectively inhibits the PTP1B enzyme (IC_50_ = 6.4 µM), acting as a competitive inhibitor capable of enhancing the insulin-induced translocation of the glucose transporter 4 (GLUT4) in CHO/GLUT4 cells [78]. In addition, the structure of the furocoumarin bound to RXRα has been solved by X-ray crystallography, as represented in Figure 7 (PDB: 3PCU). The targeting of RXRα with rutamarin triggers an increased expression and translocation of GLUT4. The compound behaves as a heterodimer-selective agonist which activates in PPARγ:RXRα dimerization [78].

**Figure 8:**
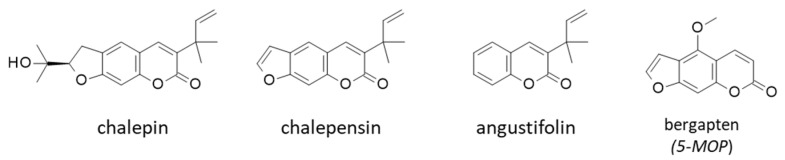
Structure of four coumarins found in *R. angustifolia* Pers.

We cannot evoke the acetyl ester rutamarin without citing the corresponding alcohol chalepin, both present in different *Ruta* species [79]. The dihydrofuranocoumarin chalepin can be isolated from the leaves *R. angustifolia* Pers. together with its furanocoumarin analogue chalepensin [13] (Figure 8). They are present in different Rutaceae plants [6]. They bear a 3-prenyl side chain, as in rutamarin. Chalepin displays a moderate antibacterial activity against *B. substilis* (Gram positive) [80] and a weak antiparasitic activity against *Trypanosoma cruzi*, the pathogen responsible for the Chagas disease. Chalepin and related coumarins can bind to the active site of *T. cruzi* glycosomal glyceraldehyde-3-phosphate dehydrogenase (gGAPDH) [81,82,83]. However, the binding affinity is weak and no anti-trypanosomal activity has been reported. Chalepin is better known for its anticancer properties. The compound displays antiproliferative activities, notably toward A549 lung cancer cells (IC_50_ = 27.6 μM), and triggers apoptosis with specific alterations of mitochondrial activities [13]. For a long time, the compound is known to reduce mitochondrial respiration, acting as a rotenone-like inhibitor of mitochondrial complex I [84]. It inhibits markedly the pyruvate/malate-supported proton flux in rat liver mitochondria (but is about 10 times less potent than rotenone) [85]. In cancer cells, chalepin not only alters mitochondria but affects also cell cycle progression and triggers extrinsic apoptosis [86,87].

A few other coumarins have been isolated from the aerial parts of *R. angustifolia*, such as 6,7,8-trimethoxycoumarin and scoparone [88]. The latter is a well-known antioxidant and lipid-lowering agent, considered useful to alleviate alcohol- or high-fat diet-induced liver injuries [89,90]. It has osteogenic effects, potentially useful to avoid bone demineralization [91], and displays antiproliferative effects against tumor cells [92]. Perhaps the most emblematic (but also confusing) coumarin is angustifolin (Figure 8), present in a very small amount of the aerial part of *R. angustifolia* [88]. The name *angustifolin* of the product is emblematic because it derives directly from Angustifolia, but it is confusing because the same name has been given to a totally different product, an ent-kaurane diterpenoid isolated from the aerial parts of *Isodon* species [93,94]. There are also four lignans named angustifolin A-D isolated from *Kadsura angustifolia* [95], and an alkaloid designated angustifoline (with a “e” at the end to respect the alkaloid terminology) found in *Lupinus* species [96,97]. Hence, the possible confusion between names Angustifolin from *Ruta* has been rarely cited, but it cannot be ignored [88].

Bergapten (5-methoxypsoralen, Figure 8) is a furocoumarin found in many plant species (notably bergamot), including *R. angustifolia* [13]. It is a classical antioxidant and anti-inflammatory compound, well known for its hypo-lipidemic, anti-ulcer and antidiarrheal activities [98,99]. It is also considered for the treatment of ischemic stroke [100,101]. Its known phototoxicity can be an issue, but the photoactivation could be exploited to treat skin cancers [102]. Bergapten displays anticancer properties, notably as a metabolic modulator for breast and colon cancer cells [103,104].

### 3.3. Other Compounds

**Figure 9:**
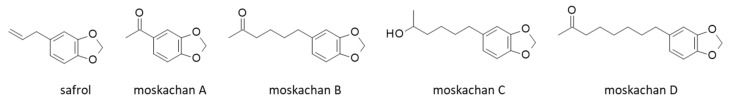
Structure of benzodioxone derivatives found in *R. angustifolia* Pers.

A relatively rare series is called moskachans A-D, four benzodioxone derivatives (Figure 9), discovered initially from the aerial part of *R. angustifolia* [105] and later found in R. chalepensis [106]. The name moskachan comes from ‘moskatxa’, the Basque name for *R. angustifolia* [105] but also used for other *Ruta* species. Moskachan B is not cytotoxic whereas moskachan D displays a weak antiproliferative activity against A549 lung cancer cells (IC_50_ = 74.4 μM) [13]. In addition to these four moskachans, other compounds with a methylenedioxyphenyl moiety have been identified from an essential oil of *R. angustifolia* (from a specimen collected in Malaysia), including compounds structurally close to safrol which is a well-known phenylpropene derivative [107] (Figure 9). Moskachans have been occasionally found in other species but rarely studied. They could be further considered as ingredients in the food or perfumery industry, as it is the case for other compounds with a methylenedioxyphenyl moiety.

## 4. Discussion

Plants of the Rutaceae family can be found in all territories worldwide. The *Ruta* L. genus originates the Mediterranean region, including the Mediterranean basin and Macaronesia, with species showing a very variable distribution. Some species are endemic to islands, such as *R. corsica* DC. in Corsica and *R. oreojasme* Webb. in the Canary Islands. *R. angustifolia* Pers. presents a circum-Mediterranean distribution, like its closest phylogenetic neighbor *R. graveolens* L. [108]. However, today, these plants can be found in all continents, including in Southeast Asia where they are even cultivated [109,110]. *R. graveolens* (garden rue, *herb-of-grace*) is far more frequently studied for its phytochemical, medicinal, ornamental properties than *R. angustifolia* which is much less known. For example, the PubMed^®^ databank includes more than 2500 references with the key word “*Ruta graveolens*” compared to less than 20 with the key word “*Ruta angustifolia*”. Nevertheless, the latter species is equally interesting in terms of phytochemical diversity and pharmacological properties, as reported here. The plant contains diverse alkaloids and coumarin derivatives of major interest, such as arborinine, graveoline, chalepin and rutamarin, and other compounds (Table 1). None of the 17 compounds listed is exclusive to *R. angustifolia* but the diversity of the products is certainly specific. Some of these compounds are very interesting from an anticancer and antiviral standpoint, such as the furocoumarin kokusaginine acting as an inhibitor of tubulin assembly in cells [37]. This compound can be found in diverse medicinal plants, not only *R. angustifolia* but also *Esenbeckia alata* Kunt [111], *Raputia heptaphylla* Pittier [112] and *Araliopsis soyauxii* Engl. [113] (three other Rutaceae species), for examples. Furoquinoline alkaloids present in *R. angustifolia* (kokusaginine, γ-fagarine) may well play a role in the activity of the plant extract in liver diseases. It is not surprising that the plant has been used traditionally to treat certain human diseases, notably liver diseases and jaundice, as mentioned in some publications [21]. However, care should be taken because the closely related furoquinoline alkaloid dictamine is notoriously hepatotoxic [114]. The bioactivity of *R. angustifolia* extracts and the traditional use of the plant probably rely on the combination of products, not on a specific product category. In Malaysia and Singapore, the local Chinese community apparently used the plant for the treatment of cancer [13]. The activity can be supported by the products like graveoline and arborinine, two alkaloids for which the anticancer action has been well established. The plant *R. angustifolia* Pers. is used traditionally to treat a variety of symptoms very often linked to inflammation. This is not entirely surprising as there are many phytoconstituents in those extracts with well-established anti-inflammatory activities, such as scoparone. This compound is a potent inhibitor of NLRP3 inflammasome activation [115]. It likely represents a key constituent of the plant extract.

Altogether, the phyto-analysis indicates that *R. angustifolia* Pers. does not present a phytochemical content less rich or less diversified compared to the better known *Ruta* species, notably the two genetically similar species *R. graveolens* L. and *R. chalepensis* L. Therefore, there is no reason to neglect this well-distributed species. In vitro cultures of the plant could be developed to increase the production of secondary metabolites, as is done with the two aforementioned *Ruta* species [116,117,118].

Edible or non-edible? The leaves of *R. graveolens* are considered as edible. They are rich in fibers (20.2%), carbohydrates (51.7%) and fats (4.2%), with a relatively protein content (10.4%) compared to other edible plants. The mineral content (notably Ca and K) is also elevated, as well as the concentration of total phenolics (1328.8 mg gallic acid equivalent (GAE)/100 g) [119]. There are no data available for *R. angustifolia* but the content is probably similar, given the close phylogenetic similarity between the two species [108]. *R. graveolens* is a renowned traditional remedy used in Ayurveda and Unani medicines, and the plant (called sudab or sadab in India) is commonly cultivated [120,121]. By analogy, it is conceivable to further exploit the leaves of *R. angustifolia* for medicinal purposes, and perhaps for food. In fact, *R. angustifolia* is listed as an edible wild plant, present in the protected area “Sierra Grande de Hornachos” in Spain. The plant is listed as a potential ingredient in the elaboration of liquors, or for dressing [122]. As a measure of precaution, it is not recommended to consume the fresh plant. *R. angustifolia* is seasonally browed by gazelles in Africa [123] but it is probably not safe for take by human, as it is the case for other rue species. The consumption of *R. graveolens* should be avoided during pregnancy, due to the risk of induced abortion [124] and the same recommendation can be drawn with *R. angustifolia* which has a quite closely similar phytochemical profile. As a general rule, *Ruta* species have long been used in traditional medicines as an abortifacient and emmenagogue [6].

As the final word of the analysis of the properties and phytochemical profile of *Ruta angustifolia* Pers. we can refer to the words pronounced by Ophelia in the play *Hamlet* from William Shakespeare (act 4, scene 5) who says “there’s rue for you; and here’s some for me”. These words could be applied to *R. angustifolia*, a little-known plant which deserves particular attention from pharmacologists and natural product chemists.

## Figures and Tables

**Table 1:** Chemical components of *R. angustifolia* and their main pharmacological activities.

Phytochemical Categories and Names	Activities	References
Alkaloids arborinine, γ-fagarine, graveoline, graveolinine, kokusaginine, pseudane IX.	Anticancer, antiviral, anti-inflammatory, antiparasitic	[21,38,39,44,49,56,57,58,59]
Coumarins angustifolin, bergapten, chalepin, chalepensin, rutamarine, scoparone, trihydroxycoumarin.	Anticancer, antioxidant, antiviral	[74,75,76,77,78,88]
Methyl ketones 2-decanone, 2-nonanone, 2-undecanone.	Antifungal, anti-inflammatory, insect repellent	[17,18,19,20,24,25]
Sterol ergosterol propyl ester.	Biocide	[23]

## Data Availability

Data sharing not applicable.
